# Awareness and attitude of first‐year medical students toward epilepsy in University of Khartoum, Sudan

**DOI:** 10.1002/brb3.2461

**Published:** 2021-12-13

**Authors:** Mohammed Elsir Ibrahim, Elfatih A. Hasabo, Elmuiz A. Hsabo, Alhadi S. Alhadi

**Affiliations:** ^1^ Faculty of Medicine University of Khartoum Khartoum Sudan; ^2^ Department of Urology Wythenshawe hospital Manchester University NHS Foundation Trust Manchester UK

**Keywords:** attitude, awareness, epilepsy, medical students, Sudan

## Abstract

**Purpose:**

Very little is known about the knowledge and attitude of students about epilepsy at Sudanese universities. Therefore, we aimed to assess knowledge and attitude of undergraduate medical students toward epilepsy.

**Methods:**

A 35‐items standardized questionnaire was self‐administered to 320 students between December 2020 and February 2021 with a recorded response rate of 61.8%. Google form was used to collect the data. The data was analyzed using R software.

**Results:**

Overall, our findings showed a negative trend in the awareness and attitude towards epilepsy. While the majority of students (84.8%) had read or heard about epilepsy, only 43.9% of them had seen someone with epilepsy. Epilepsy was considered contagious and psychological by 1.7% and 56%, respectively. About 62.2% of students believed head / birth trauma is a cause of epilepsy. On the other hand, 15.7 % and 5.1 % of students thought evil eye and divine retribution are also causes of epilepsy, respectively. The latter beliefs were more uniform among participants from rural background (*p* < .05). Regarding attitude, 19.7% of students considered it inappropriate for a patient with epilepsy to have a child. This attitude correlates with the mother's education as the percentage was higher for students whose mothers had a lower education (pre‐college education) (*p* < .05). The majority of students were aware that certain people with epilepsy need long‐term drug treatment; this belief was more uniform among females than in males (*p* < .05). The vast majority of students (93.9%) thought that a child with epilepsy could succeed in a normal class. Compared to the corresponding group, this belief was found more common in students whose mothers had a higher education (university level and above) (*p* < .05).

**Conclusion:**

This study concludes that Sudanese undergraduate students' understanding of epilepsy was minimal, necessitating a well‐directed educational campaign to develop a well‐informed and tolerant society.

## Abstract 2

This study found a low understanding of students toward epilepsy. A well‐directed educational campaign should be established to raise the awareness toward epilepsy.

## 1 INTRODUCTION

Epilepsy is a common chronic neurological disorder that is characterized by two or more unprovoked seizures. Seizures are involuntary movements that are caused by abnormally high electrical activity of neurons in the brain. Such an abnormal activity can result in various manifestations that range from dramatic convulsive activity of the body to a phenomenon that may end in a few seconds without even being recognized. Depending on the distribution of discharges, seizure may involve parts of the body (called partial) or the whole body (called generalized) and it may be associated with other various symptoms such as loss of consciousness, bowel/bladder incontinence, tongue‐biting, or other involuntary movements (Kasper et al., 2015).

In general, epilepsy is considered the most common neurological disorder affecting the brain after stroke. While the global prevalence of epilepsy is estimated to be between 5–10 per 1000, the figure in developing countries like Sudan may reach up to 9 per 1000 (Benamer & Grosset, 2009; Hermann et al., 1990). Unfortunately, epilepsy affects at least 50 million patients worldwide (Organization WH, 2005) with almost 89% percent of them living in the developing world without receiving proper treatment (Hermann et al., 1990). Consequently, individuals living in these developing countries should have good knowledge on epilepsy and adequate practices for handling patients with epilepsy (PWE) during seizures.

Several international studies have revealed a lack of awareness regarding epilepsy among the general population and even among healthcare professionals (Al‐Adawi et al., 2001; Alaqeel & Sabbagh, 2013; Chomba et al., 2007; Jensen & Dam, 1992; Vancini et al., 2012). For that, this study aimed to assess knowledge and attitudes towards epilepsy among first‐year medical students as it is likely that most of their knowledge and attitude about diseases ‐before entering medical school‐ stems from beliefs held in their communities.

## 2 METHODOLOGY

### 2.1 Survey setting and study design

This was a facility‐based descriptive cross‐sectional study that evaluated knowledge and attitude toward epilepsy among first‐year medical students. The study was carried out over the period from December 2020 to February 2021 at the Faculty of Medicine, University of Khartoum. We included all undergraduate medical students who were studying their first year at this medical college. Students who refused to participate were excluded as well as students from other universities.

### 2.2 Instruments of investigation (data collection methods)

The data was collected from the participants using Google forms. As the study was conducted during a lockdown period due to COVID‐19 pandemic, Google forms were used to help increase the number of participants and also aid the collection of data through personalized survey.

The questionnaire was adapted from a previous study by Nural Bekiroğlu and his colleagues (Bekiroğlu et al., 2004), and we used it for its reliability and validity as it was used in previous Studies (Alamri & Al Thobaity, 2020; Alamri et al., 2018;, Alqahtani, 2015; Bekiroğlu et al., 2004; Bener et al., 1998; Hills & MacKenzie, 2002; Spatt et al., 2005). The design of the questionnaire was based on (35) questions, divided into five sections: socio‐demo characteristics of the participants (6 questions), awareness about epilepsy (10 questions), attitude toward PWE (9 questions), management of the disease (5 questions), and a last section assessing the attitude of participants toward the ability of PWE to live as a normal person (5 questions). The questionnaire had been edited by adding mothers' education as this item was considered a good proxy indicator for the socioeconomic status.

To avoid any ambiguity, the questionnaire was translated to Arabic (the first spoken language in Sudan) and then it was pilot tested with five students who were not included in the study sample. After that, the questionnaire was distributed widely by posting it on social media groups that are specific to the batch.

The questionnaire was started with an introductory statement explaining the purpose and objectives of the study.

However, students were informed about the anonymous evaluation of their data and they were informed that by submitting the questionnaire, they are considered to have agreed to participate in the study. The participation in the study was completely voluntary.

The information was collected in Excel and imported to a software program for advanced statistical analysis.

### 2.3 Data analysis

Data were analyzed using R software version 4.0.2. Whereas qualitative variables were presented as frequencies and percentages, quantitative variables were converted into mean and standard deviation. Chi‐square test and Fisher's exact test were used to assess the differences between groups. A *p*‐value less than .05 was considered statistically significant.

### 2.4 Ethical approval

This study was approved by the Department of Community Medicine at the Faculty of Medicine, University of Khartoum, Sudan. The participants were asked to give consent that they agree to participate in the study by filling the questionnaire for research purposes in the online Google form.

## 3 RESULTS

### 3.1 Medical students’ characteristics

A total of 198 students participated in the study. The overall response rate was 61.8%. In general, the mean age was 19.1 years (± 0.85 SD). The majority of respondents were single (98.5%). The number of females (52%) was slightly larger than males (48%). Regarding residence of medical students, 93.9% of students were living in urban areas where only a mere of 6.1% lived in rural areas (Table 1).

**TABLE 1:** Socio‐demographic characteristics of first year medical students

Variables	Overall, *N* = 198
**Age, mean *(SD)***	19.1(0.85)
**Gender**	
Male	95 (48%)
Female	103 (52%)
**Marital status**	
Single	195(98.5%)
Married	3(1.5%)
Divorced	0 (0%)
Widow	0 (0%)
**Residence type**	
Urban	186 (93.9%)
Rural	12 (6.1%)
**Religion**	
Muslim	198 (100%)
Christian	0 (0%)
Other	0 (0%)
**Mother education**	
No education	6 (3.0%)
Primary school education	13 (6.6%)
Secondary school education	37 (18.7%)
University education and above	142 (71.7%)

*N* (%).

### 3.2 Awareness about epilepsy

The questions and answers for this section were shown in Table 2. 85.4% of participants had read or heard about epilepsy and 28.8% of them knew a person with epilepsy. Further analysis of the data showed an association between age and prior knowledge about epilepsy. Younger participants (18 years old and less) were more likely to answer “NO” for the question “Have you ever read/heard something about epilepsy?” than older participants (19 years old and above) (*p* = .003). Older participants (19 years and above) did not show any significant difference in knowing and seeing someone with epilepsy than younger participants (Table 2).

**TABLE 2:** Awareness and knowledge of epilepsy among participants

		**Age**			**Gender**			**Residency**			**Marital status**			**Mothers' education**				
**Variables**	**Overall, *N* = 198**	**≥19**	**<19**	**p‐value** *	**Female**	**Male**	**p‐value** *	**City**	**Rural**	**p‐value** *	**Married**	**Single**	**p‐value** *	**No education**	**Primary school education**	**Secondary school education**	**University education and above**	**p‐value** *
**Awareness about epilepsy**																		
Have you ever read/heard something about epilepsy? (Yes)	169 (85.4%)	136 (89.5%)	33 (71.7%)	**.003**	87 (84.5%)	82 (86.3%)	.7	159 (85.5%)	10 (83.3%)	.7	2 (66.7%)	167 (85.6%)	.4	5 (83.3%)	10 (83.3%)	34 (91.9%)	120 (83.9%)	0.6
Do you know a person with epilepsy? (Yes)	57 (28.8%)	48 (31.6%)	9 (19.6%)	.11	29 (28.2%)	28 (29.5%)	.8	51 (27.4%)	6 (50.0%)	.11	1 (33.3%)	56 (28.7%)	>.9	4 (66.7%)	6 (50.0%)	12 (32.4%)	35 (24.5%)	**0.038**
Have you ever seen someone having a seizure? (Yes)	86 (43.4%)	67 (44.1%)	19 (41.3%)	.7	41 (39.8%)	45 (47.4%)	.3	78 (41.9%)	8 (66.7%)	.094	1 (33.3%)	85 (43.6%)	>.9	4 (66.7%)	7 (58.3%)	11 (29.7%)	64 (44.8%)	0.15
Is epilepsy a contagious disease? (Yes)	15 (7.6%)	13 (8.6%)	2 (4.3%)	.5	7 (6.8%)	8 (8.4%)	.7	14 (7.5%)	1 (8.3%)	>.9	0 (0.0%)	15 (7.7%)	>.9	1 (16.7%)	1 (8.3%)	3 (8.1%)	10 (7.0%)	0.6
Is epilepsy a hereditary disease? (Yes)	98 (49.5%)	76 (50.0%)	22 (47.8%)	.8	56 (54.4%)	42 (44.2%)	.2	91 (48.9%)	7 (58.3%)	.5	1 (33.3%)	97 (49.7%)	>.9	1 (16.7%)	4 (33.3%)	17 (45.9%)	76 (53.1%)	0.2
Is epilepsy a psychological disease? (Yes)	85 (42.9%)	68 (44.7%)	17 (37.0%)	.4	37 (35.9%)	48 (50.5%)	**.038**	78 (41.9%)	7 (58.3%)	.3	0 (0.0%)	85 (43.6%)	.3	3 (50.0%)	6 (50.0%)	13 (35.1%)	63 (44.1%)	0.7
Do all patients with epilepsy have the same symptoms? (Yes)	51 (25.8%)	39 (25.7%)	12 (26.1%)	>.9	22 (21.4%)	29 (30.5%)	.14	49 (26.3%)	2 (16.7%)	.7	1 (33.3%)	50 (25.6%)	>.9	1 (16.7%)	3 (25.0%)	11 (29.7%)	36 (25.2%)	>0.9
Is epilepsy a disease that can be treated? (Yes)	131 (66.2%)	103 (67.8%)	28 (60.9%)	.4	63 (61.2%)	68 (71.6%)	.12	122 (65.6%)	9 (75.0%)	.8	3 (100.0%)	128 (65.6%)	.6	5 (83.3%)	10 (83.3%)	22 (59.5%)	94 (65.7%)	0.4
May some seizures end in few seconds without anybody recognizing them? (Yes)	160 (80.8%)	122 (80.3%)	38 (82.6%)	.7	79 (76.7%)	81 (85.3%)	.13	153 (82.3%)	7 (58.3%)	.056	2 (66.7%)	158 (81.0%)	.5	2 (33.3%)	9 (75.0%)	30 (81.1%)	119 (83.2%)	**0.035**

*N* (%).

* Fisher's exact test; Pearson's Chi‐squared test.

57 students (28.8%) have come across an individual with epilepsy. Further analysis showed that students whose mothers had university level education and above did not know a patient with epilepsy (*p* = .038). 34.4% of students considered epilepsy a psychological disorder and, interestingly, males were more likely to hold this belief than females (*p* = .038). In addition, about 19.2 % of the participants felt that seizures are not likely to end in a few seconds without being recognized (Table 2).

### 3.3 Attitude toward PWE

82.2% of participants reckoned that it is acceptable for a patient with epilepsy to get married. This positive attitude was commoner among older participants (19 years old and above) than their younger counterparts (*p* = .016) (Table 3).

**TABLE 3:** Attitude of participants toward epilepsy

		**Age**			**Gender**			**Residency**			**Marital status**			**Mothers' education**				
**Variables**	**Overall, *N* = 198*^1^***	**≥19**	**<19**	**p‐value** *	**Female**	**Male**	**p‐value** *	**City**	**Rural**	**p‐value** *	**Married**	**Single**	**p‐value** *	**No education**	**Primary school education**	**Secondary school education**	**University education and above**	**p‐value** *
**Attitude toward PWE**																		
Do you think it is appropriate for a person with epilepsy to get married? (Yes)	165 (83.3%)	132 (86.8%)	33 (71.7%)	**.016**	89 (86.4%)	76 (80.0%)	.2	157 (84.4%)	8 (66.7%)	.12	2 (66.7%)	163 (83.6%)	0.4	6 (100.0%)	7 (58.3%)	30 (81.1%)	122 (85.3%)	0.083
Do you think it is appropriate for a person with epilepsy to have a child? (Yes)	159 (80.3%)	128 (84.2%)	31 (67.4%)	**.012**	85 (82.5%)	74 (77.9%)	.4	151 (81.2%)	8 (66.7%)	.3	2 (66.7%)	157 (80.5%)	0.5	5 (83.3%)	7 (58.3%)	27 (73.0%)	120 (83.9%)	0.086
Would you marry a person with epilepsy? (Yes)	94 (47.5%)	72 (47.4%)	22 (47.8%)	>.9	57 (55.3%)	37 (38.9%)	**.021**	92 (49.5%)	2 (16.7%)	**.027**	1 (33.3%)	93 (47.7%)	>0.9	3 (50.0%)	2 (16.7%)	19 (51.4%)	70 (49.0%)	0.2
Would you approve of your child's sharing activities with a friend with epilepsy? (Yes)	161 (81.3%)	124 (81.6%)	37 (80.4%)	.9	88 (85.4%)	73 (76.8%)	.12	154 (82.8%)	7 (58.3%)	.051	2 (66.7%)	159 (81.5%)	0.5	5 (83.3%)	7 (58.3%)	29 (78.4%)	120 (83.9%)	0.15
Would you approve if your son/daughter married a person with epilepsy? (Yes)	112 (56.6%)	84 (55.3%)	28 (60.9%)	.5	66 (64.1%)	46 (48.4%)	**.026**	108 (58.1%)	4 (33.3%)	.094	2 (66.7%)	110 (56.4%)	>0.9	2 (33.3%)	5 (41.7%)	20 (54.1%)	85 (59.4%)	0.4
Do you think a person with epilepsy can live alone? (Yes)	36 (18.2%)	29 (19.1%)	7 (15.2%)	.6	16 (15.5%)	20 (21.1%)	.3	35 (18.8%)	1 (8.3%)	.7	0 (0.0%)	36 (18.5%)	>0.9	1 (16.7%)	5 (41.7%)	1 (2.7%)	29 (20.3%)	**0.005**
Would you offer a job to a person with epilepsy, if you were a boss? (Yes)	156 (78.8%)	121 (79.6%)	35 (76.1%)	.6	83 (80.6%)	73 (76.8%)	.5	146 (78.5%)	10 (83.3%)	>.9	2 (66.7%)	154 (79.0%)	0.5	6 (100.0%)	8 (66.7%)	28 (75.7%)	114 (79.7%)	0.4
Do you find it acceptable to work with a person with epilepsy (colleague) at work? (Yes)	185 (93.4%)	143 (94.1%)	42 (91.3%)	.5	98 (95.1%)	87 (91.6%)	.3	174 (93.5%)	11 (91.7%)	.6	3 (100.0%)	182 (93.3%)	>0.9	5 (83.3%)	10 (83.3%)	35 (94.6%)	135 (94.4%)	0.2
Do you think society discriminates against people with epilepsy? (Yes)	129 (65.2%)	97 (63.8%)	32 (69.6%)	.5	75 (72.8%)	54 (56.8%)	**.018**	122 (65.6%)	7 (58.3%)	.8	3 (100.0%)	126 (64.6%)	0.6	3 (50.0%)	8 (66.7%)	24 (64.9%)	94 (65.7%)	0.9

*N* (%).

* Fisher's exact test; Pearson's Chi‐squared test.

When it comes to attitudes toward marrying someone with epilepsy, 47% of students were found to be in favor of this. Such a positive attitude was more prevalent among female participants than in males (*p* = .021) and especially among those living in urban areas (*p* = .027) (Table 3).

About 56.6% of students would approve for their son/daughter to marry a PWE. Females were more likely to approve this type of marriage than their counterparts (*p* = .026). Moreover, almost two thirds (65.2%) of students thought their society discriminates against people with epilepsy and this concept was higher among females than males (*p* = .018) (Table 3).

### 3.4 Participants’ response for management of epilepsy

The vast majority of participants (96.5%) realized that some individuals with epilepsy need life‐long drug treatment. This finding was statistically higher among female participants than males (*p* = .005). Furthermore, around one third of students (29.8%) believed that smelling eau de cologne or onion could help halt an epileptic seizure (Table 4).

**TABLE 4:** Participants responses for management of epileptic seizure

		**Age**			**Gender**			**Residency**			**Marital status**			**Mothers' education**				
**Variables**	**Overall, *N* = 198*^1^***	**≥19**	**<19**	**p‐value** *	**Female**	**Male**	**p‐value** *	**City**	**Rural**	**p‐value** *	**Married**	**Single**	**p‐value** *	**No education**	**Primary school education**	**Secondary school education**	**University education and above**	**p‐value** *
**The management of disease**																		
Are there some people with epilepsy who need life‐long drug treatment? (Yes)	191 (96.5%)	146 (96.1%)	45 (97.8%)	>.9	102 (99.0%)	89 (93.7%)	.057	180 (96.8%)	11 (91.7%)	.4	3 (100.0%)	188 (96.4%)	>.9	6 (100.0%)	11 (91.7%)	35 (94.6%)	139 (97.2%)	.5
Does every person with epileptic seizure have to use an antiepileptic drug? (Yes)	92 (46.5%)	68 (44.7%)	24 (52.2%)	.4	46 (44.7%)	46 (48.4%)	.6	85 (45.7%)	7 (58.3%)	.4	3 (100.0%)	89 (45.6%)	.1	2 (33.3%)	5 (41.7%)	18 (48.6%)	67 (46.9%)	>.9
May smelling eau de cologne or onion help to end an epileptic seizure? (Yes)	59 (29.8%)	49 (32.2%)	10 (21.7%)	.2	21 (20.4%)	38 (40.0%)	**.003**	55 (29.6%)	4 (33.3%)	.8	1 (33.3%)	58 (29.7%)	>.9	2 (33.3%)	4 (33.3%)	9 (24.3%)	44 (30.8%)	.9
Do you think it is sensible to hold the arms and legs during a seizure with convulsions? (Yes)	71 (35.9%)	56 (36.8%)	15 (32.6%)	.6	36 (35.0%)	35 (36.8%)	.8	67 (36.0%)	4 (33.3%)	>.9	1 (33.3%)	70 (35.9%)	>.9	1 (16.7%)	4 (33.3%)	15 (40.5%)	51 (35.7%)	.8
Do you know how to help a patient with epilepsy during the seizure? (Yes)	52 (26.3%)	41 (27.0%)	11 (23.9%)	.7	32 (31.1%)	20 (21.1%)	.11	49 (26.3%)	3 (25.0%)	>.9	0 (0.0%)	52 (26.7%)	.6	5 (83.3%)	5 (41.7%)	6 (16.2%)	36 (25.2%)	**.005**

*N* (%).

* Fisher's exact test; Pearson's Chi‐squared test.

### 3.5 Responses regarding social activities

The vast majority of participants (93.9%) perceived that a child with epilepsy could succeed in a normal class, while 77.3% felt that PWE could succeed in some high‐ranking professions. The majority of students 82.8% regarded that PWE could participate in social activities and a similar percentage (80.3%) considered that PWE should not be prevented from participating in sports activities. There was no evidence that gender, age, or mothers’ education had influenced these beliefs (Table 5).

**TABLE 5:** Participants' responses for employment and social activities of patients with epileptic seizures

		**Age**			**Gender**			**Residency**			**Marital status**			**Mothers' education**				
**Variables**	**Overall, N = 198*^1^***	**19 ≤**	**19 >**	**p‐value** *	**Female**	**Male**	**p‐value** *	**City**	**Rural**	**p‐value** *	**Married**	**Single**	**p‐value** *	**No education**	**Primary school education**	**Secondary school education**	**University education and above**	**p‐value** *
**Attitude of participants toward the ability of PWE to live as a normal person**																		
Can a person with epilepsy be successful in some specific professions (executive secretary, physician, scientist) as ordinary people? (Yes)	153 (77.3%)	118 (77.6%)	35 (76.1%)	.8	80 (77.7%)	73 (76.8%)	.9	146 (78.5%)	7 (58.3%)	.15	2 (66.7%)	151 (77.4%)	.5	5 (83.3%)	7 (58.3%)	26 (70.3%)	115 (80.4%)	.2
Can a child with epilepsy be successful in a normal class? (Yes)	186 (93.9%)	142 (93.4%)	44 (95.7%)	.7	95 (92.2%)	91 (95.8%)	.3	176 (94.6%)	10 (83.3%)	.2	3 (100.0%)	183 (93.8%)	>.9	5 (83.3%)	11 (91.7%)	32 (86.5%)	138 (96.5%)	**.043**
Should a person with epilepsy drive? (Yes)	16 (8.1%)	12 (7.9%)	4 (8.7%)	.8	8 (7.8%)	8 (8.4%)	.9	15 (8.1%)	1 (8.3%)	>.9	0 (0.0%)	16 (8.2%)	>.9	1 (16.7%)	2 (16.7%)	4 (10.8%)	9 (6.3%)	.2
Do you think they should not often participate in social activities? (Yes)	34 (17.2%)	26 (17.1%)	8 (17.4%)	>.9	18 (17.5%)	16 (16.8%)	>.9	32 (17.2%)	2 (16.7%)	>.9	0 (0.0%)	34 (17.4%)	>.9	1 (16.7%)	3 (25.0%)	7 (18.9%)	23 (16.1%)	.8
Is it necessary for a student with epilepsy to be prevented from participating in sports activities? (Yes)	39 (19.7%)	30 (19.7%)	9 (19.6%)	>.9	21 (20.4%)	18 (18.9%)	.8	37 (19.9%)	2 (16.7%)	>.9	1 (33.3%)	38 (19.5%)	.5	1 (16.7%)	3 (25.0%)	9 (24.3%)	26 (18.2%)	.8

*N* (%).

* Fisher's exact test; Pearson's Chi‐squared test.

### 3.6 Knowledge about the etiology of epilepsy

The most commonly reported causes of epilepsy in this study were head/birth trauma (62.6%), brain tumors (58.9%), and genetics (53%). High fever was thought to be the cause of epilepsy by nearly a third of the students (35.9%). Females were more likely to hold this notion than males (*p *= .001) (Table 6).

**TABLE 6:** Knowledge of first‐year students about the etiology of epilepsy

		**Age**			**Gender**			**Residency**			**Marital status**			**Mothers' education**				
Variables	**Overall, N = 198*^1^***	**≥19**	**<19**	**p‐value** *	**Female**	**Male**	**p‐value** *	**City**	**Rural**	**p‐value** *	**Married**	**Single**	**p‐value** *	**No education**	**Primary school education**	**Secondary school education**	**University education and above**	**p‐value** *
What are cause(s) of epilepsy?																		
Genetics	105 (53.0%)	86 (56.6%)	19 (41.3%)	.069	58 (56.3%)	47 (49.5%)	.3	101 (54.3%)	4 (33.3%)	.2	2 (66.7%)	103 (52.8%)	>.9	2 (33.3%)	4 (33.3%)	13 (35.1%)	86 (60.1%)	**.013**
Medications	78 (39.4%)	63 (41.4%)	15 (32.6%)	.3	43 (41.7%)	35 (36.8%)	.5	75 (40.3%)	3 (25.0%)	.4	2 (66.7%)	76 (39.0%)	.6	3 (50.0%)	6 (50.0%)	8 (21.6%)	61 (42.7%)	.074
Head/ birth trauma	124 (62.6%)	99 (65.1%)	25 (54.3%)	.2	67 (65.0%)	57 (60.0%)	.5	119 (64.0%)	5 (41.7%)	.13	1 (33.3%)	123 (63.1%)	.6	4 (66.7%)	7 (58.3%)	20 (54.1%)	93 (65.0%)	.6
High fever	71 (35.9%)	54 (35.5%)	17 (37.0%)	.9	48 (46.6%)	23 (24.2%)	**.001**	66 (35.5%)	5 (41.7%)	.8	3 (100.0%)	68 (34.9%)	**.045**	4 (66.7%)	5 (41.7%)	15 (40.5%)	47 (32.9%)	.3
Depression or anxiety	58 (29.3%)	43 (28.3%)	15 (32.6%)	.6	37 (35.9%)	21 (22.1%)	**.033**	55 (29.6%)	3 (25.0%)	>.9	1 (33.3%)	57 (29.2%)	>.9	2 (33.3%)	5 (41.7%)	10 (27.0%)	41 (28.7%)	.8
Jinns (evil spirit)	31 (15.7%)	24 (15.8%)	7 (15.2%)	>.9	12 (11.7%)	19 (20.0%)	.11	26 (14.0%)	5 (41.7%)	.024	0 (0.0%)	31 (15.9%)	>.9	1 (16.7%)	4 (33.3%)	5 (13.5%)	21 (14.7%)	.3
Evil eye	37 (18.7%)	30 (19.7%)	7 (15.2%)	.5	15 (14.6%)	22 (23.2%)	.12	33 (17.7%)	4 (33.3%)	.2	0 (0.0%)	37 (19.0%)	>.9	0 (0.0%)	3 (25.0%)	6 (16.2%)	28 (19.6%)	.7
Punishment from God	10 (5.1%)	8 (5.3%)	2 (4.3%)	>.9	3 (2.9%)	7 (7.4%)	.2	7 (3.8%)	3 (25.0%)	**.016**	0 (0.0%)	10 (5.1%)	>.9	0 (0.0%)	0 (0.0%)	0 (0.0%)	10 (7.0%)	.4
Accidents	58 (29.3%)	47 (30.9%)	11 (23.9%)	.4	37 (35.9%)	21 (22.1%)	**.033**	55 (29.6%)	3 (25.0%)	>.9	0 (0.0%)	58 (29.7%)	.6	2 (33.3%)	4 (33.3%)	14 (37.8%)	38 (26.6%)	.5
Brain tumor	116 (58.6%)	94 (61.8%)	22 (47.8%)	.091	64 (62.1%)	52 (54.7%)	.3	111 (59.7%)	5 (41.7%)	.2	1 (33.3%)	115 (59.0%)	.6	2 (33.3%)	6 (50.0%)	24 (64.9%)	84 (58.7%)	.5
Stroke	67 (33.8%)	50 (32.9%)	17 (37.0%)	.6	37 (35.9%)	30 (31.6%)	.5	66 (35.5%)	1 (8.3%)	.063	0 (0.0%)	67 (34.4%)	.6	1 (16.7%)	4 (33.3%)	11 (29.7%)	51 (35.7%)	.8
Metabolic disease	25 (12.6%)	20 (13.2%)	5 (10.9%)	.7	19 (18.4%)	6 (6.3%)	**.01**	24 (12.9%)	1 (8.3%)	>.9	0 (0.0%)	25 (12.8%)	>.9	1 (16.7%)	2 (16.7%)	4 (10.8%)	18 (12.6%)	.8
I don't know	39 (19.7%)	24 (15.8%)	15 (32.6%)	**.012**	20 (19.4%)	19 (20.0%)	>.9	36 (19.4%)	3 (25.0%)	.7	0 (0.0%)	39 (20.0%)	>.9	0 (0.0%)	1 (8.3%)	10 (27.0%)	28 (19.6%)	.4

*N* (%).

* Fisher's exact test; Pearson's Chi‐squared test.

We also found that about 15.7% of participants thought that epilepsy could be caused by evil spirits and that participants from rural areas were statistically more likely to express this belief (*p* = .024). Likewise, 5.1% of participants viewed epilepsy as a punishment from god for wrong deeds and this false belief was found to be more prevalent by eight times in participants from rural areas as compared to those residing in cities (*p* = .016) (Table 6).

On the other hand, 29.3% (*n* = 58) of students thought that accidents could lead to the development of epilepsy. This notion was found to be more prevalent among females (*p* = .033). In addition, 12.1% of students thought that epilepsy could be a result of metabolic disorders and this was commoner among females (*p* = .01) (Table 6).

## 4 DISCUSSION

This cross‐sectional study showed a poor understanding of epilepsy in addition to inadequate practices for handling patients during seizures among first‐year medical students. Due to the prejudices and the stigma surrounding epilepsy, patients' quality of life is often significantly affected and ‐therefore‐ people in close contact with these patients should be aware of their disease.

In our study, 85.4% of students had heard or read about epilepsy. This figure is comparable to the findings of similar studies conducted in Saudi Arabia (Obeid et al., 2012) and Malaysia (Ab Rahman, 2005), 81.8% and 86.5% respectively. When compared to more recent studies ‐however‐, we found that our figure was lower than those reported in other studies conducted among German medical students (96.7%) (Mewes et al., 2020), Indian students (92.5%) (Panda et al., 2011), and Saudi students (95.3%) (Alomar et al., 2020).

Medical students, whose mothers’ education was low, which might reflect lower socioeconomic status, were likely to come across individuals with epilepsy. In a study that had reached the same conclusions, it was suggested that epilepsy is more common among the lower socioeconomic population (Ngugi et al., 2010).

Our findings have revealed that around 43.2 % of students had personally witnessed a seizure at least once in their lifetime. This percentage was relatively lower compared to the percentages reported among Jordanian (48.7 %) (Hijazeen et al., 2014), Saudi (53.3%) (Alomar et al., 2020), and Malaysian (55.6%) students (Ab Rahman, 2005).

In our study, 19.7% of students believed that PWE must not have children. This result was higher than that reported previously by studies conducted among university students in turkey (7.3%) (Alqahtani, 2015), Jordan (17.3%) (Hijazeen et al., 2014), Canada (11%) (Young et al., 2002), and Kuwait (12.5%) (Al‐Rashed et al., 2009).

In addition to this, half of the students were against marrying someone with epilepsy and this was similar to the figure quoted by a Jordanian study (50.5%) (Hijazeen et al., 2014). In studies conducted among preclinical Turkish and Nigerian students (Ekeh & Ekrikpo, 2015; Kartal, 2016), higher rates were recorded with 74.5 % and 89.3 % of them, respectively, refusing to marry someone with epilepsy.

In our study, 19.7% and 16.7% of students felt that it was inappropriate for PWE to have children or get married, respectively. A relatively similar conclusions were reached in turkey (17.3% and 16.2%, respectively) (Kartal, 2016) and slightly lower percentages were reported in Yemen (14.0% and 11.8%, respectively) (Al‐Eryani et al., 2015).

Also, we found that 81.3% and 56.6 % of students would allow their children to share activities with a friend with epilepsy or support them to marry a PWE, respectively. This positive attitude was higher than that reported in Uganda (68% and 14%, respectively) (Bigelow et al., 2015).

Our study also found 74.2% of students were aware that all PWE do not have the same symptoms and this was similar to the percentage reported among Saudi students (72.1 %) (Alomar et al., 2020).

The present study showed that 26.3% of participants reported adequate knowledge on how to help a PWE during seizure activity. This percentage was higher than that been reported in Malaysia (19.7 %) (Ab Rahman, 2005) and lower than percentages reported by Taifs’ teachers (73%) (Alamri & Al Thobaity, 2020) and Tabuk's teachers (84.2%) (Alamri et al., 2018).

We found one third of students thought that smelling eau de cologne or onion could help stop an epileptic seizure. This percentage is very low when compared with percentages that reported by Taif's teachers (71%) (Alamri & Al Thobaity, 2020) and Tabuk's teachers (74.5%) (Alamri et al., 2018).

In this study, 77.3% of students thought that a PWE can be successful in some high‐ranking professions. This was relatively similar to a study conducted at King Abdulaziz University (KAU) where 80.2% of preclinical medical students thought patients with epilepsy can be competitive in some high‐ranking professions (Kartal, 2016), but lower than those reported by Taif's (47 %) (Alamri & Al Thobaity, 2020) and Tabuk's (46.1%) teachers (Ab Rahman, 2005).

In this study, the most common reported causes of epilepsy were head/birth trauma (62.2%), brain tumor (58.9%), and genetic (52.5%). Our students and students from other countries such as Jordan (Hijazeen et al., 2014), Kuwait (Al‐Rashed et al., 2009), Saudi Arabia (Alomar et al., 2020), Turkey(26), Nigeria (Ekeh & Ekrikpo, 2015), and Yemen (Al‐Eryani et al., 2015) reported head and birth trauma as the most commonly recorded cause of epilepsy.

For many decades, epilepsy has been believed to be a disease that results from evil spirits or supernatural powers (Eadie & Bladin, 2001; Obeid et al., 2012). In this study, only 15.7% and 18.2% of students believed that epilepsy could be caused by evils spirit and evil eye, respectively. In contrast, a considerable number of Jordanian students believed that epilepsy could be caused by the evil spirit (31.5%) and evil eye (28.1%) (Hijazeen et al., 2014). These numbers were nearly similar to results in a Kuwaiti study (24.6% and 34.1%, respectively) (Al‐Rashed et al., 2009). In a Saudi study, “possession by Jinn” was reported as a cause of epilepsy by a half of students (Obeid et al., 2012). The lowest percentage was reported by Malaysian students in which only 5.3% of them thought that epilepsy could be caused by “evil spirits” (Ab Rahman, 2005).

Very few numbers of our medical students thought that epilepsy is a punishment from God (5.1%). Higher rates were reported among Yemenis (18%) (Al‐Eryani et al., 2015) and Kuwaiti (17%) students (Al‐Rashed et al., 2009). The highest rate was reported among Jordanian (26%) (Hijazeen et al., 2014) and Nigerian (26%) students (Ekeh & Ekrikpo, 2015).

To the best of our knowledge, this is the first ever study to evaluate the awareness and attitudes toward epilepsy among university students in Sudan. It raised the importance of structured epilepsy education at all levels of education in order to stop the spread of false beliefs and misleading information from public sources.

This study had several limitations that included the restriction to online data collection only using Google forms and the lack of face‐to‐face interaction which could invite social desirability bias.

## 5 CONCLUSION

Unfortunately, the findings of the study provided poor knowledge about epilepsy, negative attitudes, and incorrect management of the disease. Poor knowledge of our students could be justified by the younger age and early enrollment in medical school. We anticipate that their knowledge, attitudes, and management of epilepsy will improve significantly over time and during clinical practice later in their career.

A well‐directed educational program should be established to raise the awareness toward epilepsy among general population.

## CONFLICT OF INTEREST

None of the authors has any conflict of interest to disclose

## FUNDING INFORMATION

This project was not funded.

## AUTHOR CONTRIBUTIONS

M.E.I.: Proposal writing, questionnaire development, data collection, and writing the manuscript. E.A. Hasabo: Statistical analysis plan, data analysis and interpretation, writing the manuscript, and manuscript revision. E.A. Hsabo: Drafting the manuscript and manuscript revision. A.S.A.: Data collection and writing the manuscript. All authors reviewed and approved the final version of the paper.

### PEER REVIEW

The peer review history for this article is available at https://publons.com/publon/10.1002/brb3.2461

## Data Availability

The datasets used and/or analyzed during the current study are available from the corresponding author on reasonable request.
